# Randomized phase II study comparing the efficacy and safety of SOX versus mFOLFOX6 as neoadjuvant chemotherapy without radiotherapy for locally advanced rectal cancer (KSCC1301)

**DOI:** 10.1186/s12885-020-07766-5

**Published:** 2021-01-05

**Authors:** Keisuke Miwa, Eiji Oki, Masanobu Enomoto, Keisuke Ihara, Koji Ando, Fumihiko Fujita, Masahiro Tominaga, Shinichiro Mori, Goro Nakayama, Mototsugu Shimokawa, Hiroshi Saeki, Hideo Baba, Masaki Mori, Yoshito Akagi

**Affiliations:** 1 Multidisciplinary Treatment Cancer Center, Kurume University Hospital, Kurume, Japan; 2 Department of Surgery and Science, Graduate School of Medical Sciences, Kyushu University, Maidashi 3-1-1, Higashi-ku, Fukuoka, 812-8582 Japan; 3 Gastrointestinal and Pediatric Surgery, Tokyo Medical University, Tokyo, Japan; 4 First Department of Surgery, Dokkyo Medical University, Tochigi, Japan; 5 Department of Surgery, Kurume University School of Medicine, Kurume, Japan; 6 Department of Gastroenterological Surgery, Hyogo Cancer Center, Nishinomiya, Japan; 7 Department of Digestive Surgery, Breast and Thyroid Surgery, Kagoshima University, Kagoshima, Japan; 8 Department of Gastroenterological Surgery, Graduate School of Medicine, Nagoya University, Nagoya, Japan; 9 Cancer Biostatistics Laboratory, Clinical Research Institute, National Hospital Organization Kyusyu Cancer Center, Fukuoka, Japan; 10 Department of Biostatistics, Yamaguchi University Graduate School of Medicine, Ube, Japan; 11 Department of Gastroenterological Surgery, Gunma University Graduate School of Medicine, Maebashi, Japan; 12 Department of Gastroenterological Surgery, Kumamoto University Graduate School of Medicine, Kumamoto, Japan

**Keywords:** Rectal cancer, Neoadjuvant chemotherapy, Chemoradiotherapy, SOX, mFOLFOX6

## Abstract

**Background:**

Preoperative chemoradiotherapy (CRT), the current standard of care for locally advanced rectal cancer (LARC), is associated with many radiotherapy (RT)-related side effects. We aimed to evaluate whether S-1 and oxaliplatin (SOX) or folinic acid, 5-FU, and oxaliplatin (mFOLFOX6) can be as effective as neoadjuvant chemotherapy (NAC) regimens for LARC without RT.

**Methods:**

Patients with untreated resectable LARC were randomly assigned to receive SOX or mFOLFOX6. The NAC protocol period was 3 months. The primary endpoint was 3-year disease-free survival (DFS), and the secondary endpoints included pathological effects, surgical completion rate, 3-year survival, and safety.

**Results:**

From September 2013 to October 2015, 56 and 54 patients were enrolled in the SOX and mFOLFOX6 arms, respectively. The 3-year DFS rates were 69.4% (95% confidence interval [CI] 54.9–83.6) and 73.4% (95% CI 58.7–83.6) in the SOX and mFOLFOX6 arms, respectively; no significant differences were found between the arms (log-rank test; *P* = 0.5315, hazard ratio: 0.808, 95% CI 0.414–1.578). The 3-year survival rates were 92.3 and 91.8% in the SOX and mFOLFOX6 arms, respectively. The surgical completion rate was 98.1% overall, 100% in the SOX arm, and 96.0% in the mFOLFOX6 arm. The incidences of pathological response rates ≥grade 1b were 41.5 and 43.8% in the SOX and mFOLFOX6 arms, respectively. Both treatments were manageable and tolerable.

**Conclusion:**

We demonstrated the effectiveness and safety of SOX and mFOLFOX6, both of which may be new neoadjuvant treatment candidates in previously untreated LARC cases.

**Trial registration:**

Date of enrolment of the first participant to the trial: 3rd Oct 2013; This study was registered in the UMIN clinical trials registry on 14th Aug, 2013. (Prospectively registered, UMIN-CTR number UMIN000011486). https://upload.umin.ac.jp/cgi-open-bin/ctr/ctr.cgi?function=brows&recptno=R000013441&language=J

## Background

The incidence of colorectal cancer (CRC) is high worldwide, and the disease is third commonly occurring cancer of the United States, with rectal cancer accounting for approximately 30% of all CRCs [1]. Western countries have employed various treatment approaches for rectal cancer. The Swedish Rectal Cancer Trial showed significantly longer survival durations in association with preoperative short course radiation therapy (RT) than surgery alone [2, 3]. In a meta-analysis performed by Camma et al., who compared surgery alone with preoperative RT, the latter was associated with significantly prolonged survival durations and higher cancer-specific survival rates and significantly reduced local recurrence rates [4]. The efficacies of preoperative RT and preoperative chemoradiotherapy (CRT) were compared and reviewed in the EORTC trial 22,921, [5] FFCD 9203 trial, [6] and Polish 9203 trial [7]. Although none of the aforementioned studies showed that preoperative CRT was superior in terms of survival, the degree of downsizing, downstaging, and histological changes was significantly stronger after preoperative CRT, accompanied by a significant decrease in the rate of local recurrence. Based on the results of those clinical studies, Western guidelines recommend the use of preoperative RT or CRT for T3/T4 or N+ middle or low rectal cancer [8, 9]. However, although these preoperative treatments significantly improve local recurrence rates, no improvements in survival have been observed [10–13]. Bosset et al. reported 5 year local recurrence rates of 10.9 and 10.7% and 5 year distant recurrence rates of 32.1 and 29.8% in the CRT and CRT + adjuvant chemotherapy arms, respectively [14]. These results suggest that the suppression of distant metastasis is important for improving the survival of patients with locally advanced rectal cancer (LARC). Therefore, recently, the total neoadjuvant therapy (TNT)—characterized by the addition of systemic chemotherapy before or after CRT—has been focused to enhance the effectiveness of perioperative treatment [8, 15]. However, AEs, such as intestinal dysfunction, defecation dysfunction, sexual dysfunction, and secondary cancer occurrence, have been reported after preoperative RT. [16, 17] The toxicities caused by RT remain important concerns in such settings. A recent phase III trial examined the potential for the elimination of RT due to the effects of preoperative chemotherapy [18].

Several studies focusing on the use of preoperative chemotherapy with oxaliplatin-related regimens for rectal cancer have reported high radical resection rates (84–100%) [19–21]. Although the elimination of RT is expected to reduce the adverse event rates, there is a lack of sufficient data on the related outcomes. In particular, the use of preoperative chemotherapy, including S-1, an oral fluorinated pyrimidine for LARC, has not been investigated so far. In many clinical trials, chemotherapy using S-1 has demonstrated therapeutic results that are similar to those of chemotherapy using 5-fluorouracil (5-FU) for metastatic CRC [22–24]. We consider S-1 to be a fluorinated pyrimidine alternative to 5-FU with respect to patient preference and convenience. Therefore, we conducted a multicenter randomized phase II trial (KSCC1301) to evaluate whether S-1 and oxaliplatin (SOX) or folinic acid, 5-FU, and oxaliplatin (mFOLFOX6) are effective neoadjuvant chemotherapy (NAC) regimens without RT for LARC.

## Methods

### Patients

This trial adhered to the consort statement. This is a multicenter, open-label, randomized phase II trial. The inclusion criteria were as follows: presence of rectal or anal cancer with a low tumor border below the peritoneal reflection; histologically proven rectal anal adenocarcinoma; clinical T3–4 stage disease; clinical N0–2 disease; and M0 (TNM classification, 7th edition, 2010); however, the lymph node in the inferior mesenteric artery region was defined as N3 according to the Japanese Classification of Colorectal Carcinoma [25]; age ≥20 years; Eastern Cooperative Oncology Group performance status score 0–1; no prior chemotherapy or RT; ability to take medicine; and presence of adequate organ function. Patients were ineligible for participation if they had other primary tumors within the previous 5 years; serious cardiac disease; neurological disease; and renal, hepatic, or bone marrow dysfunction.

Signed informed consent form was obtained from all patients before enrollment to this study. The scientific and ethical aspects of the study were reviewed and approved by the institutional review board (IRB) in each participating institution. The study was conducted according to the principles of the Declaration of Helsinki, and was registered in the UMIN Clinical Trials Registry (UMIN000011486).

### Randomization and masking

Patients were assigned randomly to (1:1) SOX or mFOLFOX6 arm at the data center of the Clinical Research Support Center Kyushu using the minimization method. Stratification factors were lymph node metastasis (N0 versus N1 and N2), depth of invasion (T3 versus T4) and institution. Of the series of treatments for LARC, including NAC, surgery, and adjuvant chemotherapy, the protocol treatment in this study was NAC. All patients received the protocol treatment within 14 days of enrollment in the study.

### Procedure

In the SOX arm, S-1 + oxaliplatin was administered every 3 weeks (130 mg/m^2^ oxaliplatin on day 1 and 80–120 mg oral S-1 on days 1–14). S-1 was administered orally at dosages according to the body surface area (BSA) (120 mg/day for BSA ≥1.50m^2^; 100 mg/day for BSA 1.25–1.50m^2^; 80 mg/day for BSA <1.25m^2^). The dosage was divided into two daily doses after meals. In the mFOLFOX6 arm, 5-FU and oxaliplatin were administered every 2 weeks (85 mg/m^2^ oxaliplatin, 200 mg/m^2^ leucovorin on day 1, followed by a 400 mg/m^2^ bolus of 5-FU and a 46-h 2400 mg/m^2^ 5-FU infusion).

Before surgery, patients were scheduled to receive four cycles in the SOX arm and six cycles in the mFOLFOX6 arm. However, imaging tests were performed after two cycles in the SOX arm and three cycles in the mFOLFOX6 arm for the evaluation of whether the tumor had grown and whether radical resection was possible. If chemotherapy could be continued, two cycles were added to the SOX arm and three cycles to the mFOLFOX6 arm. Toxicity was evaluated before the start of each cycle, according to the National Cancer Institute Common Terminology Criteria (NCI-CTC) for Adverse Events version 4.0.

After NAC, radical resection was finally evaluated by performing imaging tests, and if surgery was possible, total mesorectal excision (TME) with lymph node dissection was performed. Regarding the implementation of lateral lymph node dissection, the participating facilities made decisions based on their own criteria. Postoperative adjuvant chemotherapy was started within 8 weeks using the same regimen as preoperative chemotherapy with four cycles in the SOX arm and six cycles in the mFOLFOX6 arm. After completing adjuvant chemotherapy, surveillance was conducted to check for recurrence.

### Statistical analysis

Three-year disease-free survival (DFS) was the primary endpoint. The secondary endpoints were pathological response rate, surgical completion rate, overall survival (OS) rate, and safety. This study assessed the efficacy and safety of NAC without RT using SOX or mFOLFOX6 for LARC and assessed comprehensively selected candidates in a phase III study. In the primary analysis, the 3-year DFS point estimates for the two treatments were calculated, and if one exceeded the other by more than 10%, it was determined to be a promising treatment. However, if the difference was lower than 10%, a decision was made about the right treatment option, which was deemed more likely to succeed by taking the toxicity of both into consideration. In this study, the 3-year DFS was set at 65% on the basis of previous large-scale clinical trials of preoperative RT for rectal cancer [5–7] and comprehensive evaluation of the treatment results at the participating facilities. To ensure that the probability of the accurate selection of the better treatment arm was ≥85% when the 3-year DFS exceeded 65% by at least 10%, the number of cases required was 46 for each arm, using Simon’s selection design. Finally, considering some withdrawals and cases that were deemed ineligible to receive preoperative chemotherapy, the target sample size was set as 110 cases, 55 cases in each arm. The registration period was 3 years, and the follow-up period was 5 years. DFS was defined as the duration from the date of surgery to the diagnosis of recurrence or any cause of death or the occurrence of a secondary cancer, whichever occurred first. OS was defined as the duration from the date of surgery until death of any cause. DFS and OS curves were created using the Kaplan–Meier method, and the 95% confidence intervals (CIs) were estimated using Greenwood’s formula. For primary endpoint, a one-sided *p* value less than 0.05 was considered significant in statistical tests. Statistical analyses were done by SAS version 9.4 software (SAS Institute, Cary, NC).

## Results

**Fig. 1:**
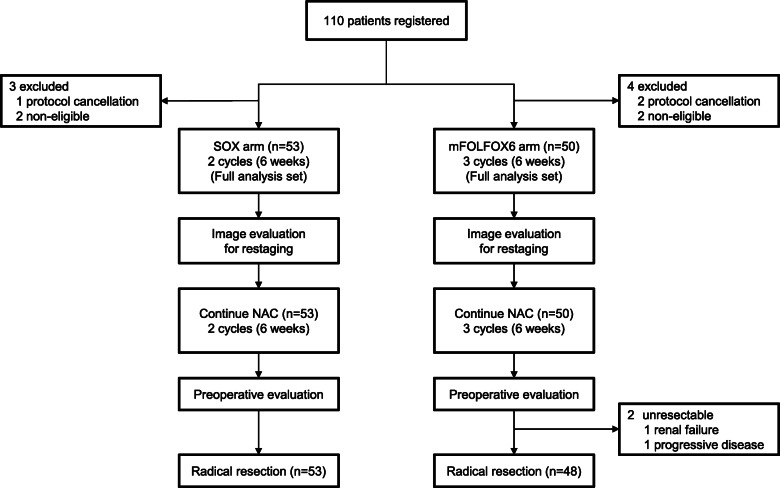
Inclusion and exclusion of patients

Overall, 110 patients were enrolled at 39 sites from September 2013 to October 2015. Although 56 and 54 patients were enrolled in the SOX and mFOLFOX6 arms, respectively, 7 patients did not meet the eligibility criteria. In total, 53 patients in the SOX arm and 50 in the mFOLFOX6 arm received NAC. Therefore, 103 patients were included in the primary endpoint analysis as the full analysis set (Fig. 1). All cases were lower rectal adenocarcinoma, and no case of anal adenocarcinoma was enrolled. The patients’ baseline characteristics at the time of registration were well balanced (Table 1). The data cutoff point was October 2018, and median follow-up for DFS, which was the primary endpoint, was 43.3 months (range, 4.2–58.3 months).

**Table 1:** Patient characteristics

Characteristics	SOX arm *n*=53 (%)	mFOLFOX6 arm *n*=50 (%)	*p*-value
Age (years)			
Median (range)	63 (37–79)	64 (36–79)	0.3440
Sex			
Male	44 (83.0)	40 (80.0)	0.6930
Female	9 (17.0)	10 (20.0)	
Performance status (ECOG)			
0	53 (100.0)	48 (96.0)	0.2332
1	0 (0.0)	2 (4.0)	
Depth of wall invasion			
T3	39 (73.6)	37 (74.0)	0.9618
T4	14 (26.4)	13 (26.0)	
Lymph node metastasis			
N0	20 (37.7)	16 (32.0)	0.6317
N1	20 (37.7)	20 (40.0)	
N2	13 (24.5)	12 (24.0)	
N3^a^	0 (0.0)	2 (4.0)	
*RAS* status			
Unexamined	37 (70.0)	36 (72.0)	0.5945
Wild	8 (15.1)	11 (22.0)	
Mutant	8 (15.1)	3 (6.0)	
Past illness/comorbidity			
Yes	23 (43.4)	23 (46.0)	1.0000
Cerebral infarction	2 (3.8)	3 (6.0)	
Myocardial infarction	1 (1.9)	0 (0.0)	
Hypertension	15 (28.3)	14 (28.0)	
Diabetes	8 (15.1)	8 (16.0)	
Others	11 (20.8)	11 (22.0)	

^a^Lymph node in the inferior mesenteric artery region,

ECOG: Eastern Cooperative Oncology Group, SOX: S-1 and oxaliplatin, mFOLFOX6: folinic acid, 5-FU, and oxaliplatin

**Fig. 2:**
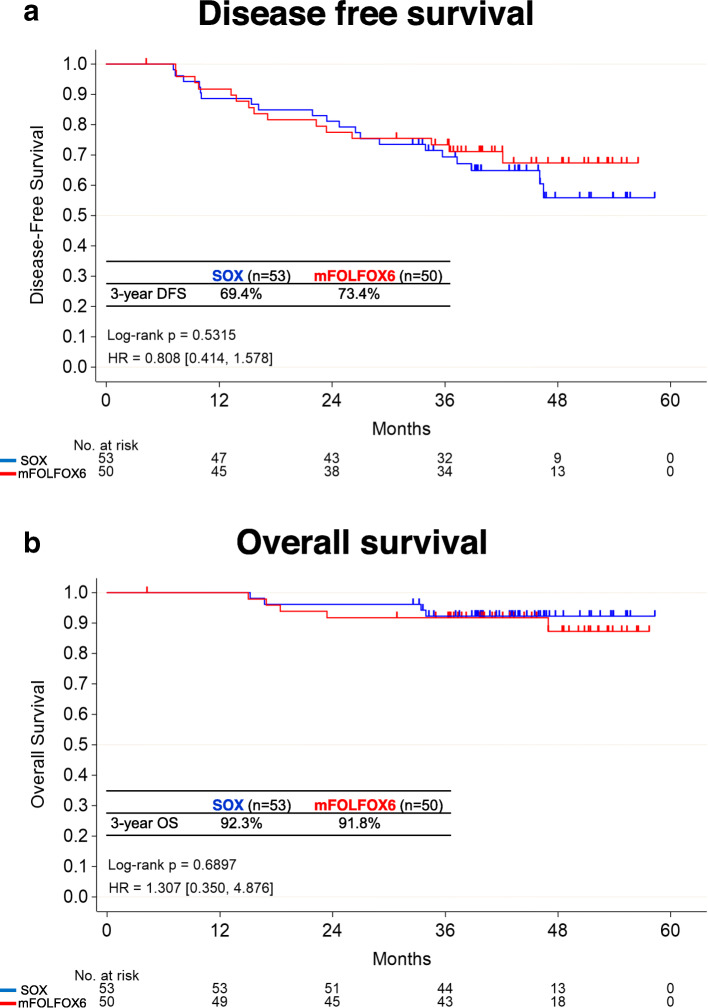
Disease-free survival (full analysis set) (**a**) and overall survival (full analysis set) (**b**)

The 3-year DFS rates 69.4% (95% CI, 54.9–83.6) and 73.4% (95% CI, 58.7–83.6) in the SOX and mFOLFOX6 arms, respectively. A significant difference was not found between the two arms (log-rank test; *p* = 0.5315; hazard ratio [HR], 0.808; 95% CI, 0.414–1.578) (Fig. 2a). The difference in the DFS rate between the SOX and mFOLFOX6 arms was 4.0% (less than 10%). The 3-year survival rates for the secondary endpoint were 92.3% (95% CI, 80.7–97.0) and 91.8% (95% CI, 79.8–96.9) in the SOX and mFOLFOX6 arms, respectively. No significant difference was found between the two arms (log-rank test; *p* = 0.6897; HR, 1.307; 95% CI, 0.350–4.876) (Fig. 2b).

A total of 101 patients underwent TME with lymphadenectomy; 31 of them underwent lateral lymph node dissection. The surgical completion rate was 98.1% (103 of 105) overall, 100% (53 of 53) in the SOX arm, and 96.0% in the mFOLFOX6 arm (48 of 50). Of the two cases in which radical resection failed, one was assessed as unresectable disease on the basis of second preoperative imaging, and the other was considered as being difficult to operate because of renal failure. The incidences of a pathological response rate of grade 1b or higher of NAC [25] were 41.5% (22 of 53) and 43.8% (21 of 48) in the SOX and mFOLFOX6 arms, respectively (Table 2). There was no significant difference between the regimens (log-rank test; *p* = 0.7442).

**Table 2:** Pathological response rate

	SOX arm	mFOLFOX6 arm	Total
	*n*=53 (%)	*n*=48 (%)	*n*=101 (%)
Grade 0	6 (11.3)	5 (10.4)	11 (10.9)
Grade 1a	24 (45.3)	22 (45.8)	46 (45.6)
Grade 1b	12 (22.6)	8 (16.7)	20 (19.8)
Grade 2	8 (15.1)	12 (25.0)	20 (19.8)
Grade 3	2 (3.8)	1 (2.1)	3 (3.0)
Unknown	1 (1.9)	0 (0.0)	1 (1.0)

SOX: S-1 and oxaliplatin, mFOLFOX6: folinic acid, 5-FU, and oxaliplatin

The incidence of AEs of all grades was 100% in both arms. The incidences of AEs of grade 3 or higher were 29.5 and 34.2% in the SOX and mFOLFOX6 arms, respectively. The AEs of grade 3 or higher that occurred in the SOX arm are shown in Table 3. The major AEs were thrombocytopenia (18.9%) and neutropenia (13.2%). In the mFOLFOX6 arm (Table 4), they were neutropenia (32.0%) and leukopenia (6.0%).

**Table 3:** Adverse events ≥grade 3 in the SOX arm (*n*=53)

	All grades	Grade 3–4
	n (%)	n (%)
Laboratory findings		
Thrombocytopenia	49 (92.5)	10 (18.9)
Neutropenia	42 (79.2)	7 (13.2)
Anemia	47 (88.7)	1 (1.9)
Hypoalbuminemia	46 (86.8)	1 (1.9)
Increased AST	42 (79.2)	1 (1.9)
Hyponatremia	23 (43.4)	1 (1.9)
Clinical findings		
Anorexia	25 (47.2)	2 (3.8)
Paresthesia	36 (67.9)	1 (1.9)
Fatigue/malaise	21 (39.6)	1 (1.9)

AST: aspartate aminotransferase, SOX: S-1 and oxaliplatin

**Table 4:** Adverse events ≥grade 3 in the mFOLFOX6 arm (*n*=50)

	All grades	Grade 3–4
	n (%)	n (%)
Laboratory findings		
Neutropenia	39 (78.0)	16 (32.0)
Leukopenia	28 (56.0)	3 (6.0)
Anemia	41 (82.0)	2 (4.0)
Hypoalbuminemia	44 (88.0)	2 (4.0)
Hyponatremia	18 (36.0)	2 (4.0)
Increased AST	38 (76.0)	1 (2.0)
Hypokalemia	14 (28.0)	1 (2.0)
Hyperkalemia	12 (24.0)	1 (2.0)
Clinical findings		
Anorexia	25 (50.0)	2 (4.0)
Catheter related		
Infection	2 (4.0)	2 (4.0)
Fatigue/malaise	23 (46.0)	1 (2.0)
Nausea	21 (42.0)	1 (2.0)
Diarrhea	21 (42.0)	1 (2.0)
Vascular disorder	1 (2.0)	1 (2.0)
Hyperglycemia	1 (2.0)	1 (2.0)

AST: aspartate aminotransferase, mFOLFOX6: folinic acid, 5-FU, and oxaliplatin

The occurrence rates of perioperative complications of grade II or higher according to the Clavien-Dindo classification [26] were 37.7 and 18.6% in the SOX and mFOLFOX6 arms, respectively (Table 5). In particular, the SOX arm had a larger number of infection-related complications, including intestinal anastomotic leakage, intra-abdominal abscess, and wound infection, than the mFOLFOX6 arm. No grade IV or higher complications according to the Clavien-Dindo classification were observed in both arms. Finally, 44 (83.0%) and 38 (79.2%) patients in the SOX and mFOLFOX6 arms, respectively, received postoperative adjuvant chemotherapy.

**Table 5:** Perioperative complications ≥grade II per the Clavien-Dindo classification

	SOX arm	mFOLFOX6 arm	Total
	*n*=53 (%)	*n*=48 (%)	*n*=101 (%)
Postoperative hemorrhage			
IIIa	0 (0.0)	1 (2.1)	1 (1.0)
Intestinal anastomotic leakage			
II	1 (1.9)	1 (2.1)	2 (2.0)
IIIa	1 (1.9)	0 (0.0)	1 (1.0)
IIIb	4 (7.5)	0 (0.0)	4 (4.0)
Intra-abdominal abscess			
II	1 (1.9)	0 (0.0)	1 (1.0)
IIIa	3 (5.7)	0 (0.0)	3 (3.0)
IIIb	2 (3.8)	0 (0.0)	2 (2.0)
Wound infection			
II	4 (7.5)	1 (2.1)	5 (5.0)
IIIa	2 (3.8)	1 (2.1)	3 (3.0)
Ileus			
IIIa	1 (1.9)	2 (4.2)	3 (3.0)
IIIb	1 (1.9)	2 (4.2)	3 (3.0)
Pneumonia			
II	0 (0.0)	1 (2.1)	1 (1.0)

SOX: S-1 and oxaliplatin, mFOLFOX6: folinic acid, 5-FU, and oxaliplatin

## Discussion

In the present study, we found that SOX and mFOLFOX6 as NAC without RT are effective and safe in LARC settings. Regarding NAC, we selected an oxaliplatin-based regimen that is effective against metastatic CRC and reportedly has a high R0 resection rate even in preoperative chemotherapy. Specifically, for convenience, we decided to compare the mFOLFOX6 regimen, widely used in metastatic CRC, with the SOX regimen, containing oral fluorinated pyrimidine. Although SOX and mFOLFOX6 have been demonstrated to show similar therapeutic effects in metastatic CRC, [24] no study to date has evaluated the therapeutic outcomes of SOX as an NAC regimen for LARC.

In this study, the 3-year DFS rates were 69.4 and 73.4% in the SOX and mFOLFOX6 arms, respectively. The 3-year DFS rates in a previous pivotal phase III trial using CRT for rectal cancer were 60–70% based on Kaplan–Meier curves, [5, 7] similar to our findings. In a recent phase III trial (FOWARC trial) conducted in China, the efficacies of infusional 5-FU + RT + TME, mFOLFOX6 + RT + TME, and mFOLFOX6 + TME were compared; the 3-year DFS rates were 72.9, 77.2, and 73.5%, respectively, and the 3-year OS rates were 91.3, 89.1, and 90.7%, respectively [27]. In terms of the 3-year DFS rate, which was the primary endpoint, no difference was noted between the three arms, but the pathological complete response rate was significantly higher in the mFOLFOX6 + RT arm [28]. In our study, the 3-year OS rates were 92.3 and 91.8% in the SOX and mFOLFOX6 arms, respectively, and the 3-year DFS and 3-year OS rates were comparable to those in the FOWARC trial. Therefore, although no difference greater than 10% was observed between both arms, we considered that the treatment outcomes were satisfactory in both arms. Further follow-up is required in the future.

The overall rate of transition to radical resection in this study was 98.1% (103 of 105), which is a satisfactory result. According to the results of two Japanese prospective studies reporting the efficacy of capecitabine + oxaliplatin + bevacizumab as NAC for LARC, the surgical completion rates were 92% (23 of 25) [8] and 84% (27 of 32) [21]. Although the characteristics of our participants were different, the surgical completion rate in the SOX arm of this study was 100% (53 of 53), suggesting the effectiveness of SOX as an NAC regimen for LARC. In addition, there was no significant difference in the pathological effectiveness between the regimens, and we believe that it is acceptable to select either as an NAC regimen.

The AEs that occurred in both the regimens were the same as those noted in previously reported clinical phase III trials [29, 30] and are within expectations. Both treatments were manageable and tolerable. In terms of perioperative complications, infection-related complications were more commonly observed in the SOX arm than in the mFOLFOX6 arm, but the cause was unknown. However, complications that were grade IV or higher according to the Clavien-Dindo classification were not observed in both arms. In addition, the transition rate to postoperative adjuvant chemotherapy was comparable between the arms (83.0% versus 79.2%). These results indicate the safety of both regimens.

In Japan, D3 dissection with lateral lymph nodes, which preserves pelvic autonomic nerve function without preoperative CRT, and subsequent adjuvant chemotherapy are widely used to improve the outcomes of patients with LARC [31]. Mizushima et al. reported that the 3-year DFS rate was 70.1% in a phase II study of 107 patients with high-risk stage II and stage III rectal cancer without preoperative treatment who had received capecitabine + oxaliplatin therapy as postoperative adjuvant chemotherapy, [32] similar to the findings of our study. There is controversy surrounding whether chemotherapy for LARC should be performed before or after TME in Japan; this issue needs to be resolved in a future phase III study.

Our study has some limitations. First, it had a phase II study design; it is necessary to plan a phase III trial that compares the efficacy of NAC with preoperative CRT or postoperative adjuvant chemotherapy. The standard treatment for LARC is CRT. The regimens used in this study are by no means a substitute for CRT. Second, the incidences of distant metastases and survival were not evaluated because of the insufficient follow-up period. Third, RT was not used. In this study, we selected an RT-free approach to avoid the side effects of RT and to determine if a recently developed chemotherapy regimen could be used in a study arm compete with future CRTs. Further, the therapeutic efficacy of chemotherapy using these regimens as TNT remains unclear. We believe that these two regimens are worth assessing as TNT for LARC in the future.

This study demonstrated the efficacy of SOX and mFOLFOX6 containing oral fluoropyrimidine as NAC regimens for resectable LARC. Data on NAC without RT for T3–T4, N0–N2 resectable rectal cancer are currently very limited. Therefore, we believe that our study is extremely valuable because it provides data on the use of NAC without RT for rectal cancer.

## Conclusion

SOX and mFOLFOX6 as NAC regimens without RT are effective and safe and may be new neoadjuvant treatment candidates in LARC settings.

## Data Availability

The datasets used and/or analysed during the current study are available from the corresponding author on reasonable request.

## Abbreviations

CRT: chemoradiotherapy
LARC: locally advanced rectal cancer
SOX: S-1 and oxaliplatin
mFOLFOX6: folinic acid, 5-FU, and oxaliplatin
NAC: neoadjuvant chemotherapy
RT: radiotherapy
DFS: disease-free survival
CRC: colorectal cancer
TNT: total neoadjuvant therapy
NCI-CTC: National Cancer Institute Common Terminology Criteria
TME: total mesorectal excision
CIs: confidence intervals (CIs)
AEs: Adverse events
